# A century of “Camel Research”: a bibliometric analysis

**DOI:** 10.3389/fvets.2023.1157667

**Published:** 2023-06-01

**Authors:** Mahmoud Kandeel, Mohamed A. Morsy, Hany M. Abd El-Lateef, Mohamed Marzok, Hossam S. El-Beltagi, Khalid M. Al Khodair, Wafaa E. Soliman, Ibrahim Albokhadaim, Katharigatta N. Venugopala

**Affiliations:** ^1^Department of Biomedical Sciences, College of Veterinary Medicine, King Faisal University, Hofuf, Saudi Arabia; ^2^Department of Pharmacology, Faculty of Veterinary Medicine, Kafrelsheikh University, Kafrelsheikh, Egypt; ^3^Department of Pharmaceutical Sciences, College of Clinical Pharmacy, King Faisal University, Hofuf, Saudi Arabia; ^4^Department of Pharmacology, Faculty of Medicine, Minia University, El-Minia, Egypt; ^5^Department of Chemistry, College of Science, King Faisal University, Hofuf, Saudi Arabia; ^6^Department of Chemistry, Faculty of Science, Sohag University, Sohag, Egypt; ^7^Department of Clinical Sciences, College of Veterinary Medicine, King Faisal University, Hofuf, Saudi Arabia; ^8^Department of Surgery, Faculty of Veterinary Medicine, Kafrelsheikh University, Kafrelsheikh, Egypt; ^9^Agricultural Biotechnology Department, College of Agriculture and Food Sciences, King Faisal University, Hofuf, Saudi Arabia; ^10^Biochemistry Department, Faculty of Agriculture, Cairo University, Giza, Egypt; ^11^Department of Anatomy, College of Veterinary Medicine, King Faisal University, Hofuf, Saudi Arabia; ^12^Microbiology and Immunology Department, Faculty of Pharmacy, Delta University for Science and Technology, Gamasa, Egypt; ^13^Department of Biomedical Sciences, College of Clinical Pharmacy, King Faisal University, Hofuf, Saudi Arabia; ^14^Department of Biotechnology and Food Science, Faculty of Applied Sciences, Durban University of Technology, Durban, South Africa

**Keywords:** bibliometrics, camels, web of science, camel, research

## Abstract

**Introduction:**

Bibliometrics is a quantitative analytic strategy used to assess the unit of publications per each field of research. Bibliometric studies are commonly employed to examine the current research climate, potential developments, and development trends in certain domains. In this work, the major contributors to camel research throughout the past century are discussed, along with the funding sources, academic institutions, scientific disciplines, and countries that contributed to “Camel Research”.

**Methods:**

The Web of Science (WOS) database was used to retrieve the publications based on the Preferred Reporting Items for Systematic Reviews and Meta-Analyses (PRISMA) instructions.

**Results:**

There are 7,593 articles dedicated to camel research on the Web of Science (as of August 1st, 2022). Three stages were involved in the publication of a study on camels. At the beginning, from 1877 to 1965, there were fewer than ten new publications per year. The second stage comprised 100 publications per year (1968–2005). Since 2010, nearly 200 new papers have been published each year. King Saud and King Faisal universities contributed > (0.08) of the total publications. While more than 1,000 funding agents were retrieved, the Natural Science Foundation of China (NSFC) showed the greatest rate of funded projects (0.17). Camel research was included in 238 scientific disciplines. The top disciplines were Veterinary Sciences (0.39), Agriculture Dairy Animal Science (0.144), and Food Science Technology (0.087).

**Conclusion:**

There has been an increase in interest in camels in recent years, but the research trends in camel health and production need greater support.

## 1. Introduction

To assess the present state of research in a field, bibliometric techniques are utilized. The bibliometric analysis makes it much simpler to determine, within a specific area of research, the research scholars, universities, papers, and search terms that receive the most attention and are most frequently cited. This is possible through the identification of the most proactive and highly cited research (1). Additionally, it allows experts to evaluate a publication's quality and coherence, and it can also manifest the author's profile. Anticipating emerging trends in different fields is one of the key benefits of this type of study (2). It is frequently used to investigate the state of existing research, future perspectives, and growth patterns of certain fields (3). Publications are one of the key means by which advances in scientific research and technological development are displayed. The extrinsic parameters of scientific publications serve as the research subjects for the quantitative analysis method known as bibliometrics (4). As per various illustrations of experimental observations, this approach can be classified into bibliographical statistical, mathematical, system, matrix and network analysis (1). Thus, the current study uses bibliometric analysis to follow the development of camel research and its effects on academia and the public across published works that use camel referencing papers included in the Web of Science (WOS) (1900–2022).

The camel is considered the most suitable domesticated animal for desert regions with sporadic and inconsistent yearly rainfall and lengthy, arid, warm seasons lasting at least 8 months (5). Camels have been domesticated for approximately 3,000-6,000 years, Old World camels have benefited humans in cross-continental migrations by carrying people and commodities, bridging cultural barriers, and producing milk, meat, and wool (6). Animal husbandry and livestock science are challenged by the expansion in deserts caused by extreme weather events and the rising demands for sustainably produced meat and dairy products (7). The dromedary camel should be taken into consideration as the most adapted and sustainable organism that could be used the harsh climate caused by global changing weather patterns that are defined by a consistent rise in desertification, high temperatures, and also water shortages. Dromedary camels are adaptable to these extreme weather changes and extremely effective in their production (8, 9). Interestingly, several recent reports have shown that the dromedary camel would be the foremost species that manage to survive as the best source of farm animals for future agribusiness and the animal production industry thanks to the advances of the desert world, primarily to actively participating in achieving the Sustainable Development Goals (9–12). Dromedaries, however, have gained attention from the research community than other farmed animals (13). In this regard, a greater awareness of the number of camel-related research studies that have been conducted and the leading camel research nations will assist to enhance the sort of research that should be conducted in the future.

Previously, very limited bibliometric studies have been conducted on camels (14, 15). One of these bibliometric studies investigates 4,923 camel scholarly articles from 1963 to 2012 (a period of 50 years), which were retrieved from the CAB Direct Online database (14). Recent reports have also traced past scientific papers indexed in the ScienceDirect directory and analyzed the impact on camel husbandry and welfare (15).

A systematic approach for tracking the advancements in an area of study and identifying the attributes of certain publications is offered by bibliometric analysis. This article offers a bibliometric study of camel-related scientific publications from 1877 to 2022. This will help in examining the state-of-the-art research products in the camel sector, including key discipline statistics, famous journal developments, and publication patterns in literature. Moreover, to take into account the representative regions, prominent authors, and most referenced publications.

## 2. Materials and methods

### 2.1. Theoretical aspects of bibliometric method

Bibliometrics is the study of quantitative methods and measures for analyzing and evaluating scholarly publications and communication. It is an interdisciplinary field that draws on information science, mathematics, statistics, and sociology. The main objective of bibliometrics is to provide objective, quantitative measures of the impact and influence of research publications, authors, and journals. The development of bibliographic databases, such as the Science Citation Index, Web of Science, Scopus and others, made it possible to analyze the patterns of citation and publication in scientific literature. Bibliometrics uses a variety of quantitative measures to assess the impact and influence of scholarly publications, such as publications count, active researchers and institutions. Bibliometric analysis is widely used in academia, research institutions, and funding agencies to assess the impact and influence of research publications and to make decisions about funding, promotions, and tenure.

### 2.2. Database search and retrieval of articles

The data collection was achieved by first identifying the databases and choosing appropriate search strategy techniques, data retrieval techniques, and cleaning the data before feeding them into different tools for analysis and visualization. For this research, WOS was used to retrieve the bibliometric data, and the data was exported to Excel. The study used Excel to provide the visual graphs for the bibliometric data on camels.

The term “camel” that was included in the title, abstract, and keywords was used to retrieve bibliographic data from the WOS database. The WOS database is the primary data source for bibliometric studies and offers extensive, multidisciplinary citation data. A large volume of data was retrieved for the years 1877 to 2022. Using bibliometric principles, the categories assessed were affiliations, authors, document type, funding, countries/regions, grant numbers, open access, publication year and WOS category. These are the major bibliometric parameters established in other research publications (16). Bibliometric analysis can be used to analyze a research trend in a particular field.

### 2.3. Eligibility assessment

**Figure 1:**
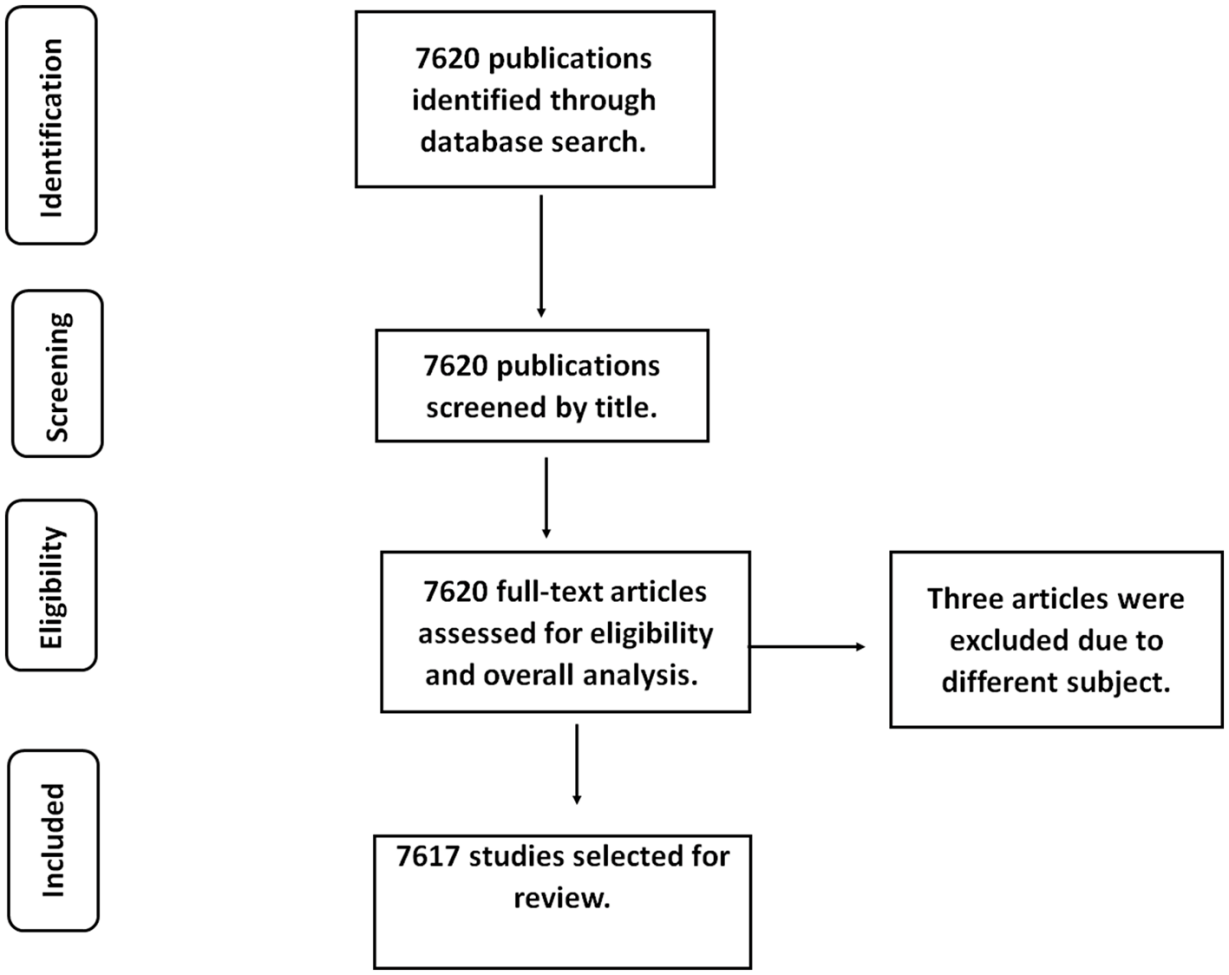
PRISMA flowchart.

Two independent reviewers assessed the eligibility of the studies included in the bibliographic analysis based on several criteria. Foremost, the study restricted the literature to those only published in English. The articles selected were first based on the relevant content provided in the title and the abstract. The exclusion criteria also involved those studies that were not using camels in the experiments. During the review, critical features extracted from the full text included the author, titles, recent citations, year of publications, institutions of research and funding agencies. Finally, any disagreement arising from the inclusion or exclusion of a study was resolved through consensus. The PRISMA flowchart for the article retrieval steps is provided in Figure 1.

### 2.4. Article inclusion/exclusion criteria

The study did not exclude any of the parameters provided in the WOS since the study intended to explore all the publications stored on camels. Retrieved documents included articles, book chapters, editorial materials, and reviews. Since only English words were employed to search for relevant publications, the search may have left out some critical articles published in other languages such as French, Spanish, and Chinese. Therefore, the results may not be generalized to other researchers published in non-English speaking countries. Although the analysis may not include all the crucial publications on camels, the researcher believes that this study's results offer a reliable insight into the trends and patterns of the publications made on camels.

## 3. Results and discussion

Using the term “camel” in the paper title, summary, or search terms, the WOS database was accessed for collecting reference information on the study subject. A total of 7,617 documents were extracted from WOS between the period 1877 and 2022. The information that was acquired provides insights into the most prominent scholars and the most active institutions in the field of camel research, which may also be helpful for the identification of funding opportunities and potential for collaboration.

### 3.1. Document types

**Figure 2:**
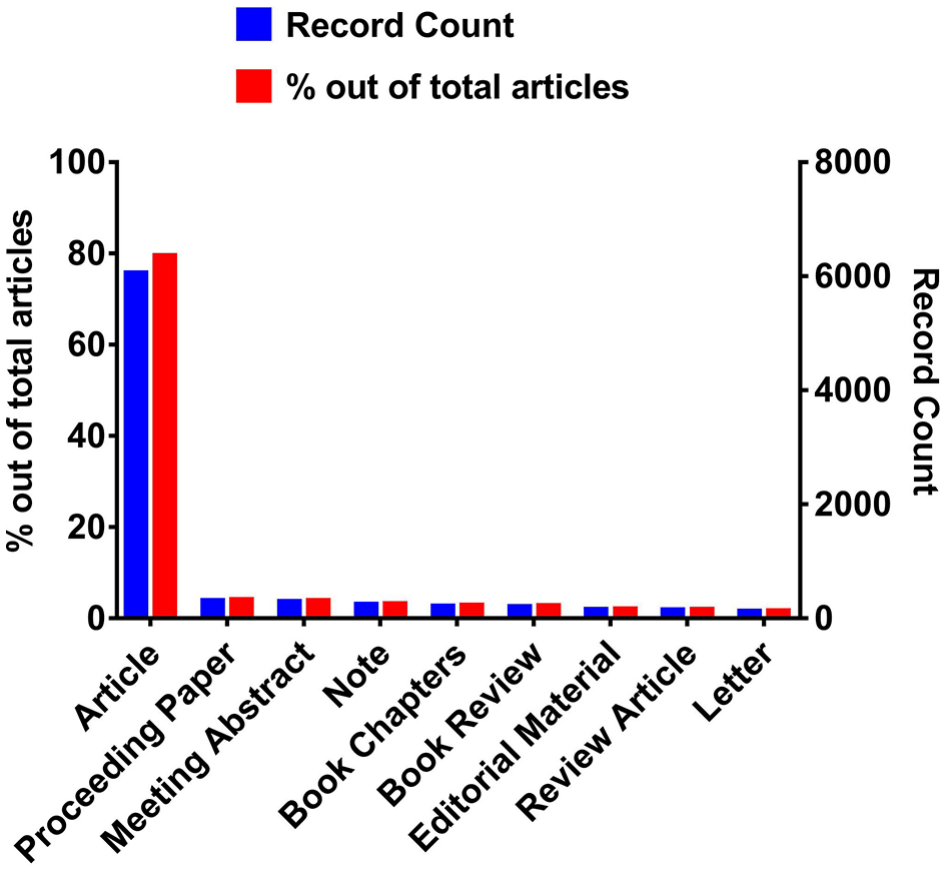
The number of document types published between 1900–2022 in the camel research field in WOS.

The type of document retrieved was analyzed based on the WOS database classification. Publications on camels were distributed in 26 publication types. Out of a total of 7,617 documents, 6,052 (0.794) documents were articles which indicate article was the most prevalent type of document and has the highest contribution to camel research during the 1877–2022 period. Furthermore, proceeding papers (312; 0.041), meeting abstracts (293; 0.038), notes (244; 0.032), book chapters (216; 0.028), book reviews (207; 0.027), Editorial material (158; 0.07), review article (145; 0.019), letter (124; 0.016), corrections (44; 0.006), news items (41; 0.00538), poetry (35; 0.00459), early access (24; 0.003), fiction, creative prose (13; 0.0017), film reviews (6; 0.00079), record reviews (5; 0.00066), books (4; 0.00053), data papers (4; 0.00053), correction, addition (3; 0.00039), excerpts (3; 0.00039), art exhibit reviews (2; 0.00026), reprints (2; 0.00026) type documents were also found. While biographical-items, dance performance reviews, hardware reviews, and software reviews were the type of documents that showed the lowest contribution (1, 0.00013) in camel research during the 1900–2022 period. Figure 2 represents the document types out of a total of 7,617 articles contributed in the camel field between 1877–2022 time interval. The observed number of open-access papers in camel research highlights the recent trends in funding agents of camel research projects to spread camel-related knowledge. The roles played by funding agencies and research organizations in the advancement of scientific inquiry are of the utmost significance (17, 18).

### 3.2. Countries/regions

**Figure 3:**
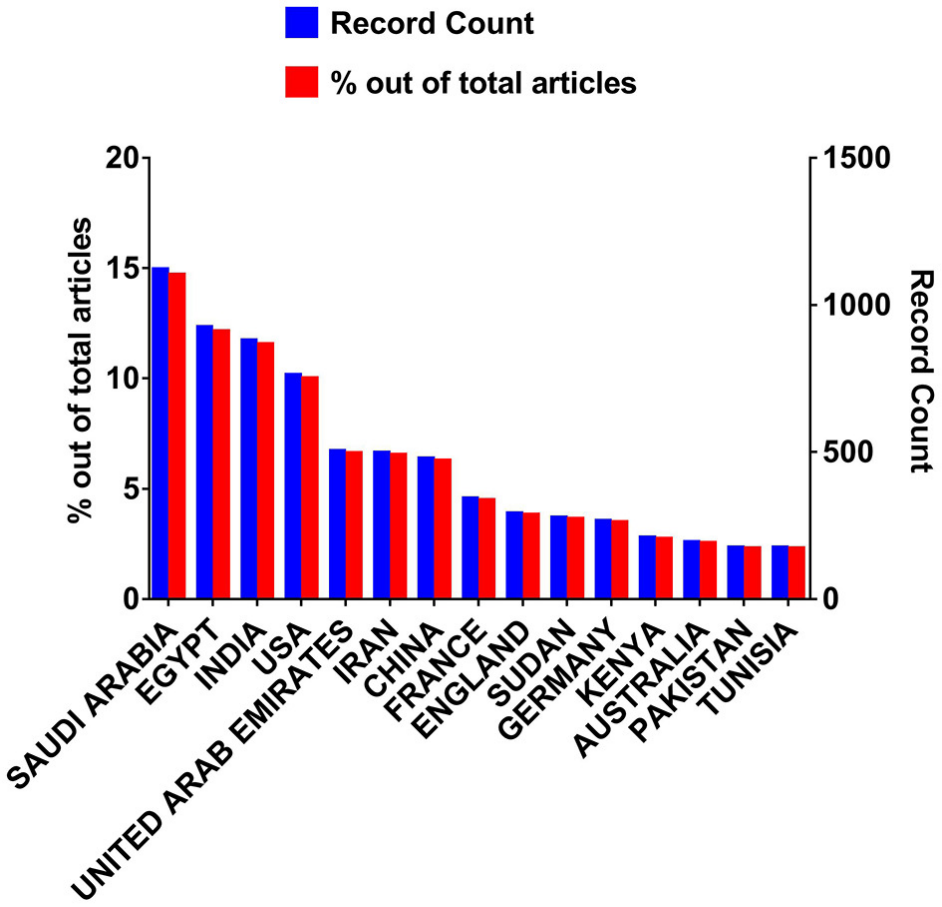
The top fifteen nations that published the most papers on camels between 1877 and 2022.

Regarding the countries, a total of 7,617 articles were published in 131 countries according to the literature database. Figure 3 shows the top 15 countries with a maximum number of publications during 1877–2022. The complete list is provided in Supplementary Table 1. These countries include Saudi Arabia, Egypt, India, USA, United Arab Emirates, Iran, China, France, England, Sudan, Germany, Kenya, Australia, Pakistan and Tunisia. Saudi Arabia appeared as the country with the highest number of publications i.e., 1,117 (0.1467) papers out of 7,617 published papers. This showed the highest contribution of Saudi Arabia in the field of camel research. Egypt ranked second with 921 (0.120) papers out of 7,617 published papers followed by India which scored in the third position due to the publication of 876 (0.115). Thus, Saudi Arabia, Egypt, and India are the top three countries with the highest number of publications related to the camel field and playing a major role in this field. The USA with 759 (0.0997) publications out of 7,617 papers stand at the fourth position, UAE at the fifth position with 500 (0.066) publications, Iran at sixth position with 494 (0.0649) publications, China at seventh position with 474 (0.06223) publications, France at eighth position with 338 (0.0444) publications, England at ninth position with 288 (0.0378) publications, Sudan at tenth position with 288 (0.0378) publications, Germany at 11th position with 262 (0.0344) publications and Kenya at 12th position with 205 (0.0269) out of 7,617 papers. While publications from Australia, Pakistan, Tunisia, Morocco, Italy, Ethiopia, Spain, Japan and Canada countries range between 100–200 i.e., 190 (0.0249), 172 (0.0225), 172 (0.0226), 153 (0.0201), 145 (0.019), 132 (0.0173), 110 (0.0144), 107 (0.014), 105 (0.0138) respectively. Moreover, Sweden, Algeria, Jordan, Belgium, Nigeria, Turkey, Oman, Kazakhstan, Netherlands, Switzerland, Austria, and Russia with publications ranging between 50–99 papers from 1900 to 2022 i.e., 98 (0.01287), 95 (0.0125), 92 (0.012), 84 (0.011), 83 (0.0109), 78 (0.01024), 77 (0.0011), 76 (0.00998), 70 (0.00919), 65 (0.00853), 54 (0.0071) and 52 (0.00683) papers, respectively. Denmark, Scotland, Iraq, Malaysia, Mongolia, Qatar, Taiwan, Kuwait, South, Africa, Fed Rep Ger, Libya, Hungary, Brazil, Ireland, Czech Republic, Somalia, South Korea, New Zealand, Poland, Norway, Syria, Thailand, Uganda, Botswana, Singapore, Mauritania, Uzbekistan published 10–49 papers during the period of 1900-2022 such as 47 (0.00617), 43 (0.00565), 41 (0.00538), 41 (0.00538), 39 (0.00512), 39 (0.00512), 38 (0.00499), 36 (0.00473), 35 (0.00459), 28 (0.00368), 27 (0.00354), 26 (0.00341), 24 (0.00315), 24 (0.00315), 23(0.003), 23 (0.003), 22 (0.0029), 20 (0.0026), 17 (0.0022), 16 (0.0021), 16 (0.0021), 14 (0.0018), 13 (0.0017), 12 (0.0016), 12 (0.0016), 11 (0.0014) and 10 (0.0013) papers respectively. Argentina and Bahrain both published nine (0.0012) papers, each of three countries i.e., Greece, Portugal and Romania published eight (0.0011) papers, each of four countries i.e., Ger Dem Rep, Lebanon, United Arab Rep, Yemen published seven (0.00092) papers, each of four countries i.e., Bangladesh, Serbia, Turkmenistan and Ussr published six (0.00079) papers, each of six countries i.e., Burkina Faso, Mali, Niger, Tanzania, Vietnam and Wales published five (0.0007) papers from 1900 through 2022. Chad, Croatia, Indonesia, Malawi, and Senegal were the countries that published four (0.0005) papers, while Chile, Ecuador, Eritrea, Mexico, and Slovenia each published three papers from 1900 through 2022. Each Azerbaijan, Bulgaria, Cambodia, Czechoslovakia, Djibouti, Ghana, Jamaica, Lithuania, Namibia, North Ireland, and Peru published only two papers related to the camel field during the 1900–2022 time period. Each of 29 countries including Afghanistan, Bosnia Herceg, Brunei, Burundi, Colombia, Costa Rica, Cuba, Dem Rep Congo, Honduras, Hong Kong, Kosovo, Latvia, Liberia, Luxembourg, Madagascar, Monaco, Mozambique, Nepal, North Korea, Palestine, Rep Congo, Slovakia, Sri Lanka, Trinidad Tobago, Uruguay, Venezuela, West Germany, Yemen Arab Rep, Yugoslavia, Zambia were the countries which published least number papers i.e., only one (0.00013) during the 1900–2022 period.

The observed findings coincide with the previous reports of camel livestock contribution by countries. Saudi Arabia has been classified as one of the countries with a high proportion of camel livestock (19). Egypt showed second rank in the list of publications, and despite Egypt's classification as a camel country, it has a declining growth with a low proportion of camel livestock (19). There is an increasing interest in camels in China in recent years. Individuals in China are becoming more interested in drinking camel milk because of the purported health benefits it offers as well as the wool and leather industry from Bactrian camels (20–22). In the USA, there are only 3,000 heads of camels distributed among US private farms (19). There is now increasing interest in camels due to the beneficial use of camel milk in treating diabetes, colitis and other somatic diseases (23). The United Arab Emirates is classified as one of the countries with an extremely lofty proportion of camel livestock (19).

### 3.3. Authors

**Figure 4:**
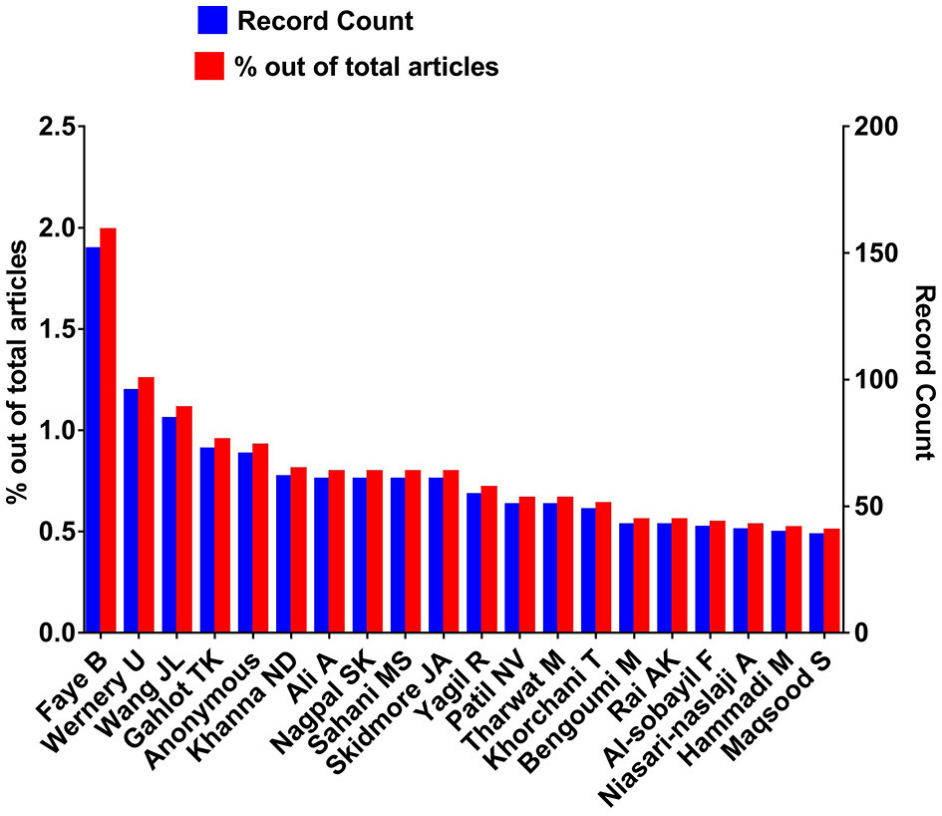
Top twenty most active camel researchers who published between 1877 and 2022.

Results from WOS illustrated that Faye B was the most productive author with 151 (0.0012) publications in the camel research field from 1877 to 2022 followed by Wernery U (95; 0.0125), Wang JL (84; 0.011), and Gahlot TK (72; 0.0095). So, these were the authors who contributed most to the camel research field. Figure 4 shows the top 20 authors with the highest number of publications searched from WOS. The complete list of authors and their contributions is provided in Supplementary Table 2.

### 3.4. Affiliations

**Figure 5:**
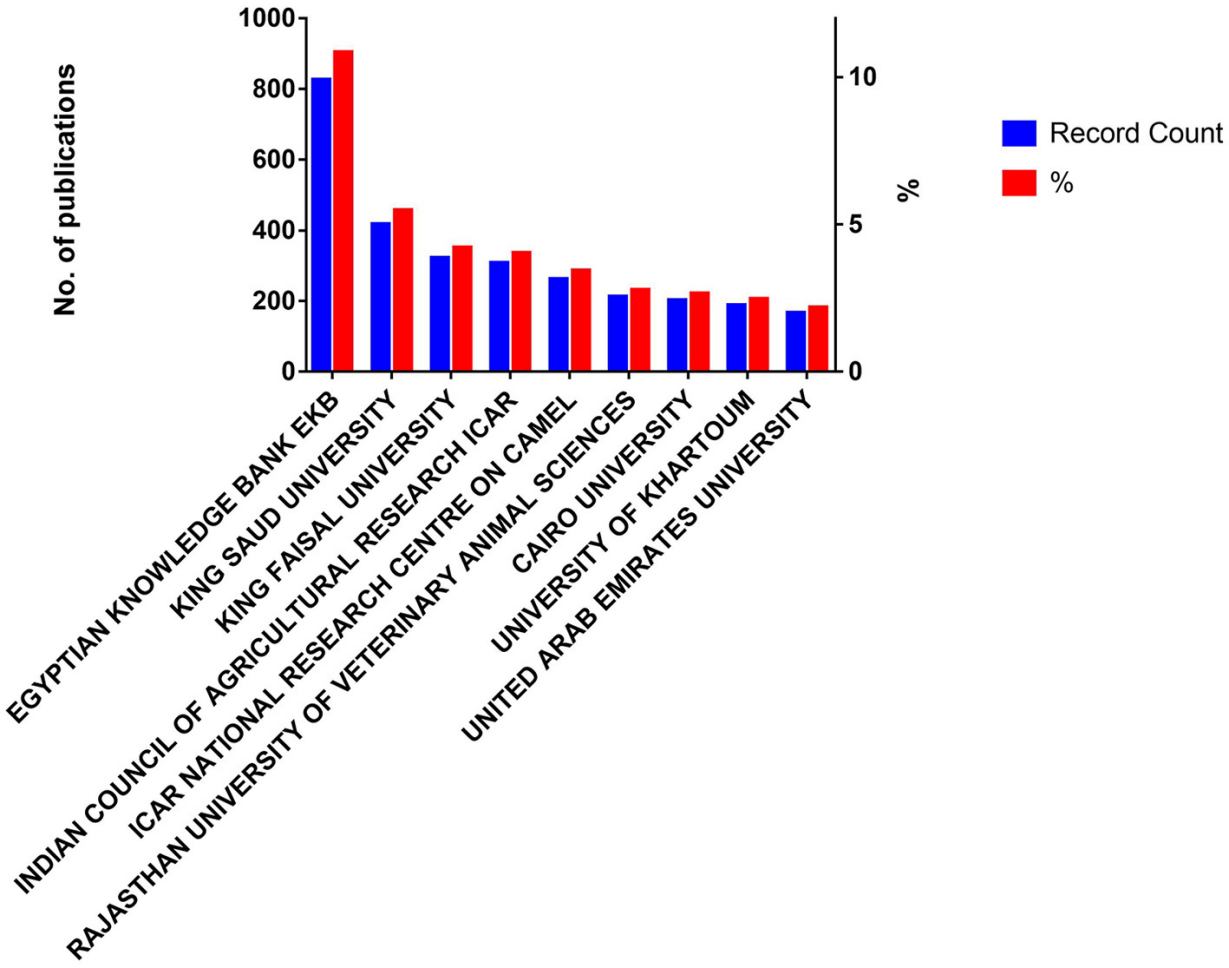
Most common affiliations of camel-related publications during 1877–2022.

Affiliation of most of the publications found with Egyptian Knowledge Bank (EKB) (824; 0.1082), followed by King Saud University (416; 0.055), King Faisal University (320; 0.042), Indian Council of Agricultural Research (306; 0.04), National Research Centre on Camel (261; 0.034), Rajasthan University of Veterinary Animal Sciences (211; 0.0277), Cairo University (201; 0.0264), University of Khartoum (187; 0.0256), United Arab Emirates University (165; 0.0217), and CCS Haryana Agricultural University (134; 0.0178). This data shows that most of the studies were conducted by authors affiliated with Saudi Arabia and Indian Universities. Figure 5 shows the most common affiliations of camel-related publications. The complete list of affiliations and their contribution to camel research is provided in Supplementary Table 3. This data is in line with the previous classification of Saudi Arabia as a country with a sharp increase in camel population following regular growth (19). This could be accompanied by increased interest in developing camel research. According to the data that is curated by the Ministry of Environment, Water, and Agriculture in Saudi Arabia, the number of camels is estimated to have more than 1.39 million heads in 2018 and has been growing annually by 0.52 since 1961. This represents a significant increase from the number of camels that existed in 1961 (24). Camel countries with a sharp increase in camel population following regular growth comprise countries within the top common affiliations.

### 3.5. Trends in scientific publications (Per-year research)

**Figure 6:**
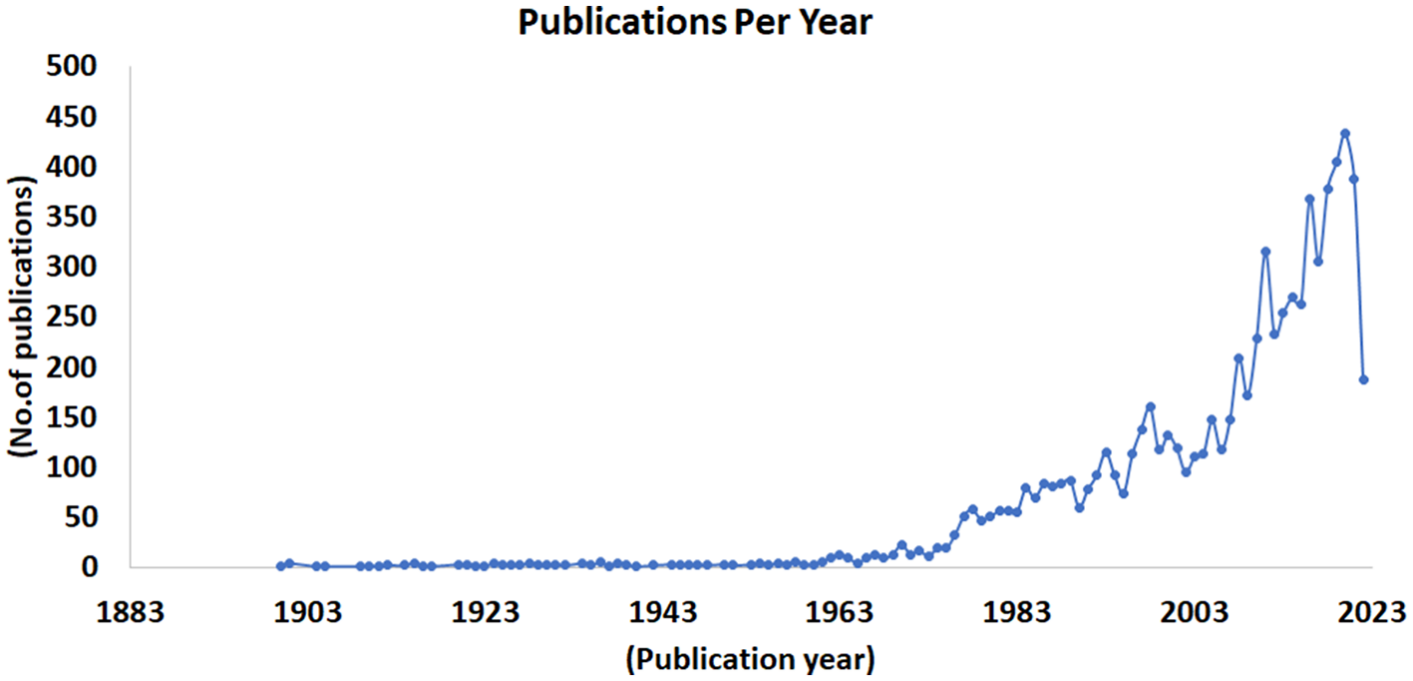
Number of articles per year in camel research published during 1877–2022.

This investigation displays camel-related research publications from 1900 through 2022 in order of publication year. Article counts by year, from 1900 through 2022, are shown in Figure 6. From the graph, we may deduce that between 1900 and 1960, annual publishing rates were rather low, falling in the range of 1–6. Annual publishing rates rose steadily from 1961 to 1999. There were over a hundred publications that were seen for the very first time in 1993 (115; 0.015). More than a hundred articles were published between 1994 and 1996. More than 100 papers each year have been made in camel research from the year 2000 until 2022. In 2020, there were 433 papers published in camel research, representing a 5.68 percent annual increase over the previous year's output. Annual publishing rates fell to 388 (0.051) in 2021. There will have been 187 papers published by August 2022, representing 0.0246 of the total. This demonstrates that 2020 was the most fruitful year for camel study. It's possible that one of the reasons for the recent uptick in camel research is that (1) there are now more camel-focused publications and special issues available. (2) In recent years, the association of camels with respiratory virus outbreaks known as MERS-CoV infection has contributed to an increase in the number of camel studies and citations for those studies. Among these are works that are relevant to MERS-CoV that have received a large number of citations in the camel scientific community. For instance, highly cited articles included 975 (25) and 411 (26) citations.

### 3.6. The most active funding agents

**Figure 7:**
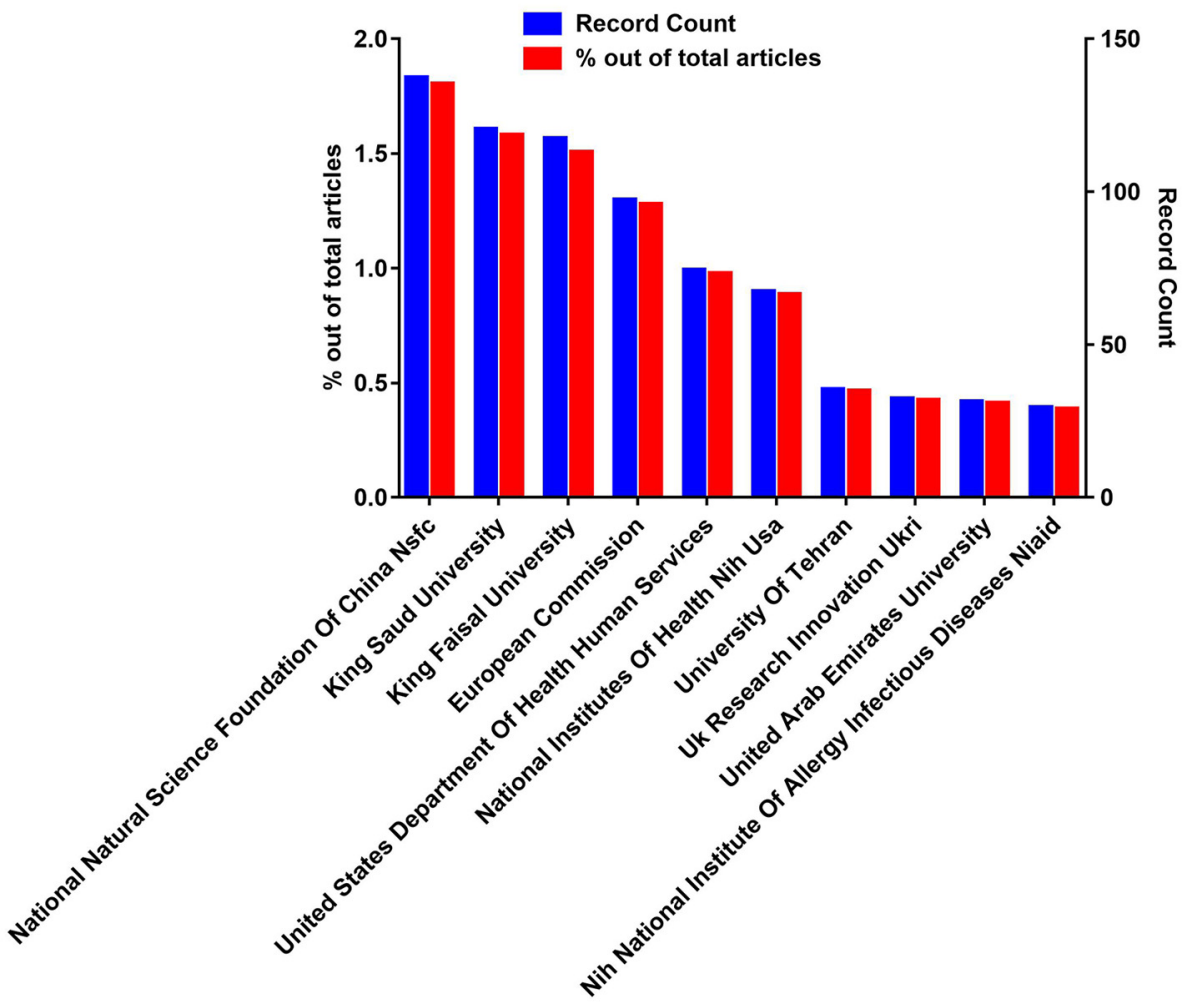
The top ten most active funding agencies in camel research.

Considering funding, out of 7,617, a total of 2,078 publications appeared in the search through WOS. The highest publications i.e., 137 (0.0179) received funding from the National Natural Science Foundation of China (NSFC) followed by King Saud University (120, 0.0158), European Commission (97; 0.0173), United States Department of Health and Human Services (74; 0.0097), and National Institutes of Health (NIH) USA (67; 0.0088). While the rest of the funding agencies provide funds to <10 publications. This shows that NSFC is one of the main funding agencies with a significant contribution to accelerating the research related to camels. Figure 7 shows the top ten funding agencies empowering camel research. The complete list of funding agents is provided in Supplementary Table 4.

### 3.7. Grant number and number of publications

Grant numbers are unique identifiers assigned to research grants by funding agencies or institutions. In bibliometric analysis, grant numbers are important because they allow researchers to track the impact of funding on scientific publications. By analyzing grant numbers, bibliometricians can identify the research projects that were funded by a particular grant or funding agency. This information can then be used to evaluate the productivity and impact of the research produced by the grant recipients. Grant numbers can also be used to identify collaborations between different institutions or researchers, as well as to identify trends in research funding over time. In addition, grant numbers can be used to identify potential conflicts of interest in research. For example, if a particular grant is awarded to a researcher who is also a member of a company's board of directors; this could raise questions about the objectivity of the research findings.

**Figure 8:**
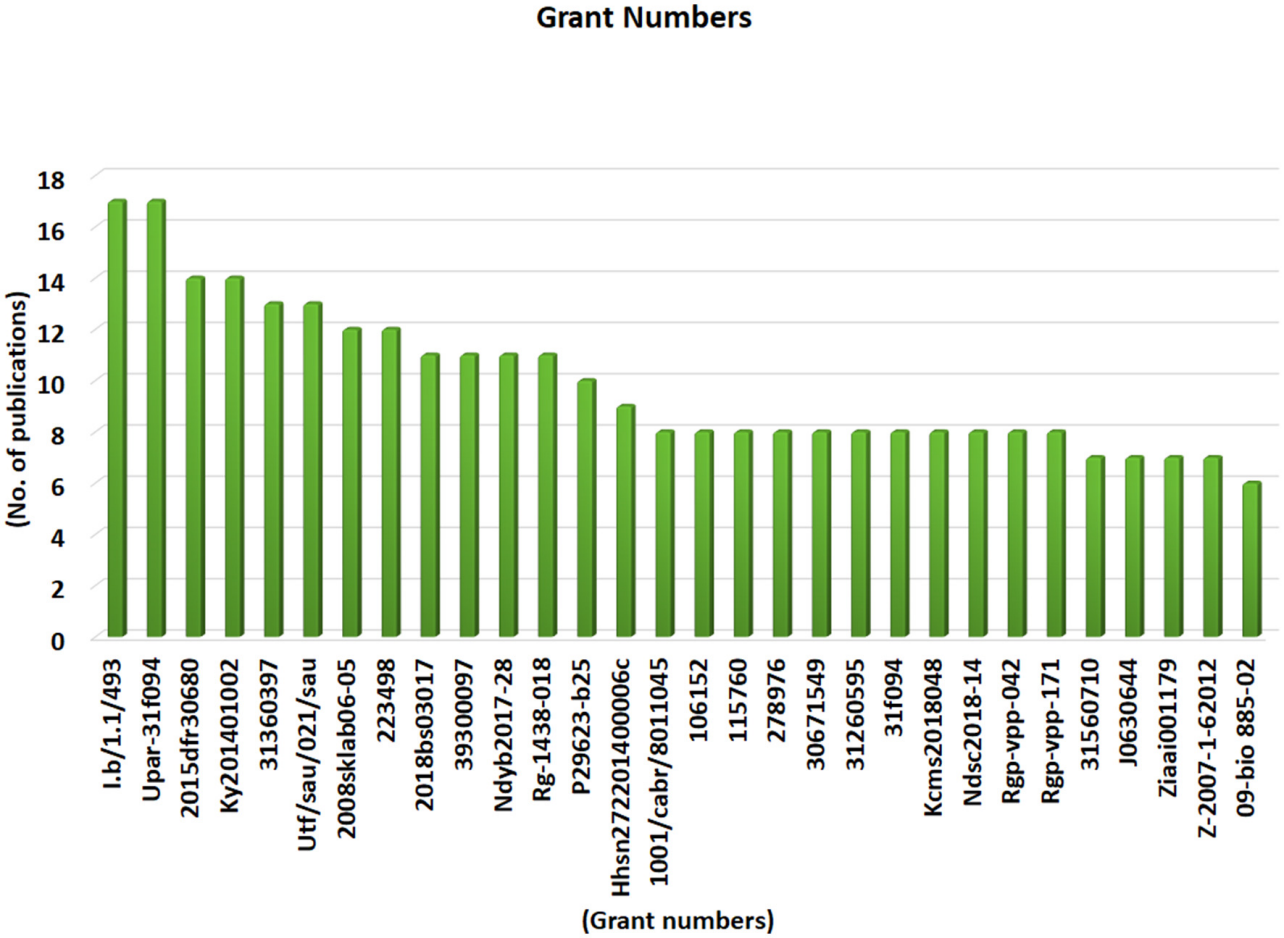
Top thirty Grant Numbers related to camel research searched from WOS between 1877–2022.

From 1877 to 2002, WOS databases turned up 1,530 grant numbers associated with camel research. I.b/1.1/493 and Upar-31f09417 grant numbers were mentioned by the highest number of publications (17; 0.233), while 2015dfr30680 and Ky201401002 grant numbers were mentioned in 14 (0.00184) publications individually. 31360397 (13; 0.0017), Utf/sau/021/sau (13; 0.0017), 2008sklab06-05 (12; 0.0016), 223498 (12; 0.00156), 2018bs03017 (11; 0.0014), 39300097 (11; 0.0014), Ndyb2017-28 (11; 0.0014), Rg-1438-018 (11; 0.0014) and P29623-b25 (10; 0.0013) were observed in more than 10 publications searched through WOS. The rest of the grant numbers were found in fewer publications. Figure 8 illustrates the top thirty Grant Numbers related to camel research.

### 3.8. Open access

Open access is very important in bibliometric analysis because it provides researchers with unrestricted access to scientific literature, which allows them to discover and use a wider range of sources in their analysis. Open-access articles are available to anyone with an internet connection, which can increase the visibility and impact of scientific research. Open access is a critical component of bibliometric analysis because it promotes greater scientific discovery and collaboration, while also enabling more accurate and comprehensive analysis of scientific literature. By making research articles freely available, open access promotes greater collaboration and exchange of ideas among researchers, which can lead to new discoveries and advances in science. It also enables research to be more easily reproduced and verified, which is an important aspect of scientific inquiry. In bibliometric analysis, open access can facilitate the identification of relevant publications for citation analysis and impact measurement. It can also reduce bias in the analysis by ensuring that all publications, regardless of their source or publisher, are available for evaluation. In this study, the fraction and distribution of open-access categories were analyzed.

**Figure 9:**
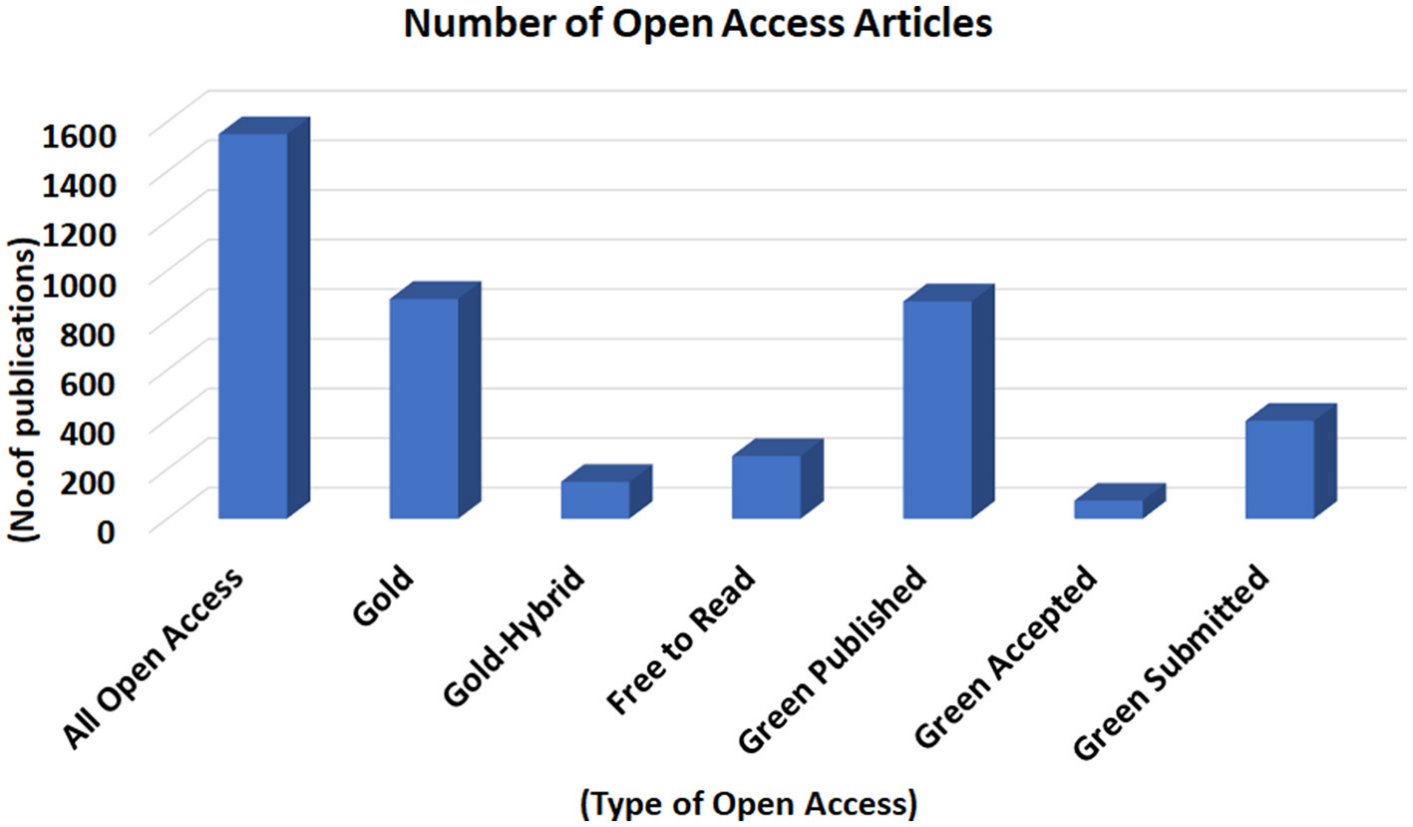
All Open Access publications in camel research during 1877–2022.

From the total of 7,617 papers in the camel research field during the 1877-2022 period, 1,550 (0.2035) papers were published as all/complete open access, 886 (0.1163) with gold open access, 149 (0.01956) with gold-hybrid open access, 253 (0.0332) were free to read, 876 (0.115) were green published, 73 (0.0096) were green accepted and 395 (0.0519) were green submitted. So, most of the articles i.e., 1,550 in the camel research field during the 1877-2022 period were published with all open access as shown in Figure 9. The rest of the documents including 6,067 (0.7971) papers were not found in the open access category.

### 3.9. WOS categories

WOS stands for Web of Science, which is a bibliographic database that indexes and provides access to scholarly literature from various fields. WOS categories refer to the subject categories or fields in which the indexed literature is classified. The importance of WOS categories lies in their ability to organize and retrieve literature based on subject matter. Researchers and academics can use these categories to search for literature relevant to their area of study, as well as to identify research trends and collaborations within specific fields. However, there are also limitations to WOS categories. For example, some articles may fall into multiple categories, making it difficult to classify them accurately. Additionally, the categories themselves may not always accurately reflect the interdisciplinary nature of modern research, leading to potential biases in how research is organized and evaluated. Furthermore, not all academic disciplines are represented equally in the WOS database, which may limit its usefulness in certain areas of study.

**Figure 10:**
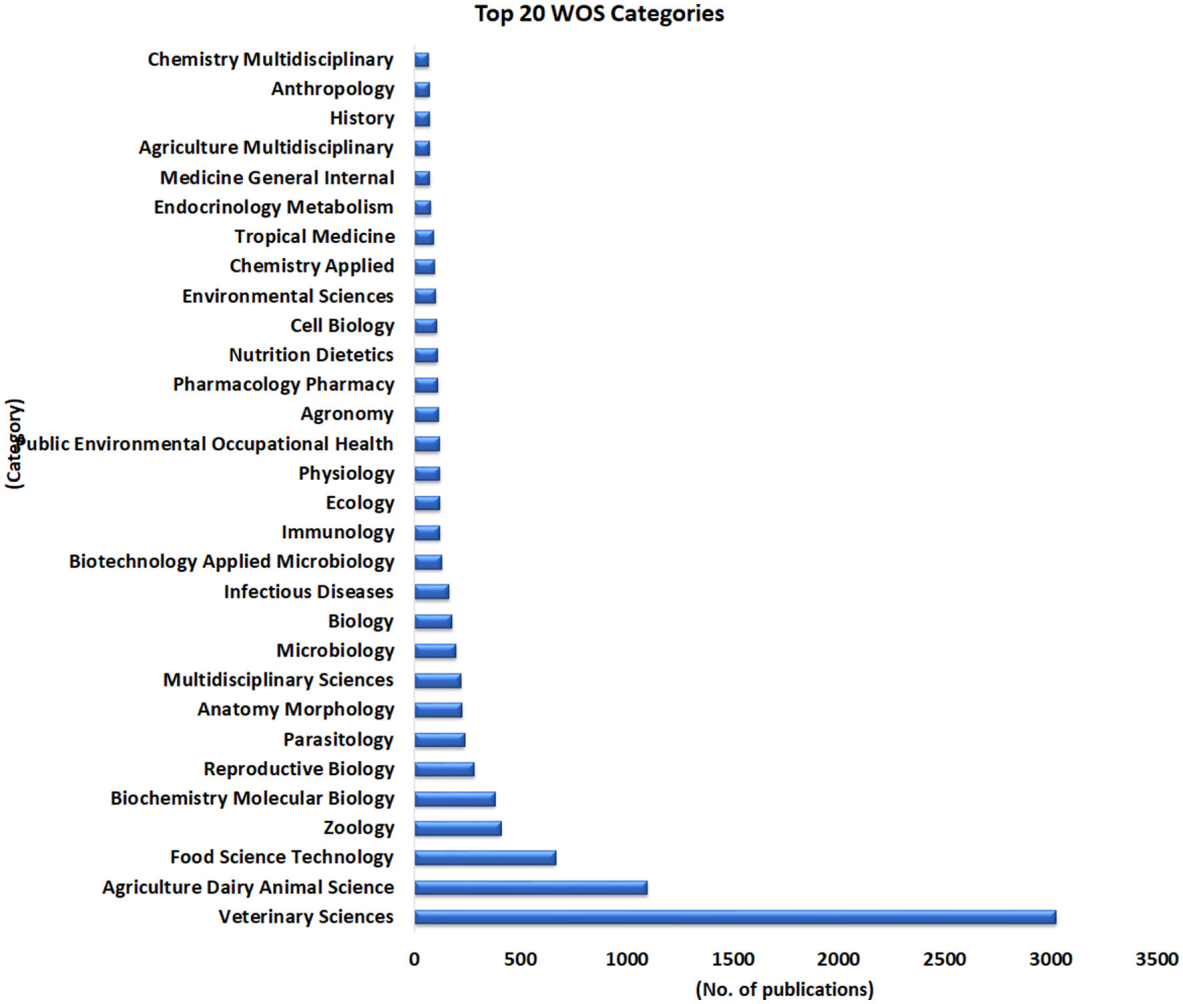
Top twenty categories of WOS with the highest number of publications in camel research during 1877–2022.

All 7,617 publications related to camel search were observed in 238 categories of WOS. Of which the highest i.e., 3,023 (0.397) publications were collected from Veterinary Sciences followed by Agriculture Dairy Animal Science (1,097; 0.144), Food Science Technology (665; 0.0873), Zoology (408; 0.0536), and Biochemistry Molecular Biology (380; 0.0499). The top twenty WOS categories are shown in Figure 10. The complete list of WOS categories is provided in Supplementary Table 5.

The bibliometric research on camels is helpful, in particular to the veterinary practitioners as well as the academics, because it provides direction on the developments that are taking place in the sector. By browsing WOS categories of camel publications, camel practitioners can gain insight into the scientific disciplines and new discoveries in their field. Both veterinary practitioners and academics in several ways: 1) Identifying research trends: This information can be used by veterinary practitioners and academics to focus their research efforts on areas that are receiving significant attention in the field. 2) Assessing the impact of research: By analyzing citation patterns, researchers can identify which studies have had the most significant influence on the field, and which have been less impactful. 3) Identifying knowledge gaps: Bibliometric analysis can also help identify areas where there is a lack of research. In addition, students can increase their knowledge of the right locations in which to seek the material that is important to the topic. Along with other approaches for the scientific visualization of mapping, the given co-author network may be able to provide insight into some of the authors who are particularly well-known in the area. In addition, there are other databases that can provide further information, such as the keywords that are used the most, referrals to publications, and citation networks. Because of this, one of the shortcomings of this study is that it only uses a single database. Nevertheless, there is a possibility that integrating many databases would provide skewed results due to the fact that part of the information may be readily replicated. On the other hand, upcoming scholars in this subject will be able to conduct analysis by retrieving data from other databases, such as Web of Science and Scopus. To further broaden the scope of bibliometrics in this area, future scholars might additionally assess the nations or institutions that have produced the most significant number of articles.

### 3.10. Limitations

A limitation of this study is that only articles that were published on the Web of Science were taken into consideration for this study. This is done to guarantee that the camel research that is published is of high quality. This might be considered a restriction as well because many additional papers are published in journals that are not included in WOS. The publications included in this work were only available in the English language, which is another limitation of this study.

## 4. Conclusions

This study used the bibliometric approach to analyze the patterns and characteristics of scientific literature in camel research, such as the number of publications, authors, journals, funding agents and scientific disciplines. Camel research studies revealed a dramatic rise in activity, funding, and participation from a wide range of countries. More scholarly publications pertaining to camels were published in 2020, roughly exceeding 200 publications per year, showing a rise in the field's profile. Because of this, researchers can now explore several previously unexplored facets of camels, including improved methods for the diagnosis, management, and prevention of infectious disease; increased milk and meat production; and, most importantly, the biological and pharmaceutical applications of camel milk. This study identified the most influential authors and institutions in camel research, which is led by several Asian and African countries. The study shed light and identified emerging research areas through the exploration of the WOS categories. There are already 238 disciplines that make use of camel research. Veterinarian medicine, dairy animal science in agriculture, and food science technology were the top three majors. Research into camel health and productivity is still needed, despite the animal's rising popularity in recent years. Continuous follow-up bibliometric studies are required to ascertain the newly added disciplines and patterns of camel-related publications.

## Data availability statement

The original contributions presented in the study are included in the article/Supplementary material, further inquiries can be directed to the corresponding author.

## Author contributions

MK, MMo, HA, MMa, WES, and HE-B planned the study design, contributed to data analysis, and wrote and revised the manuscript. MK, IA, and KV contributed to data extraction and analysis. All the authors approved the final version of the manuscript.
